# Posture control characteristics of different age groups in China based on Bertec^®^ balance advantage CDP/IVR—a cross-sectional study

**DOI:** 10.3389/fneur.2025.1658938

**Published:** 2025-11-06

**Authors:** Yaqi He, Pei Zhang, Qing Li, Zhongmei Peng, Jinpeng Gao, Yang Liu, Fan Yang, Ye Tian, Xianglin Lv, Jinghua Qian

**Affiliations:** 1 School of Sports Medicine and Rehabilitation, Beijing Sport University, Beijing, China; 2 Key Laboratory of Exercise Rehabilitation Science of the Ministry of Education, Beijing Sport University, Beijing, China; 3 Affiliated Sports Hospital of Chengdu Sport University, Chengdu, China

**Keywords:** aging, balance, postural control, rhythmic weight shift test, sensory organization test

## Abstract

**Objective:**

To establish the first normative dataset for postural control in Chinese adults using Bertec^®^ CDP and to explore potential cross-cultural differences in aging trajectories by comparing with Western population data.

**Methods:**

This cross-sectional study recruited 48 healthy Chinese adults stratified into four age decades (18–35, 36–45, 46–60, and 61–75 years). Participants attended three laboratory visits: the first two for screening and task familiarization, and the third for formal testing on the sensory organization test (SOT) and rhythmic weight shift (RWS) using computerized dynamic posturography (CDP), with 3–7 days between visits. Data were analyzed using one-way ANOVA with *post-hoc* tests. Exploratory comparisons with Danish normative data were conducted using one-sample *T*-tests.

**Results:**

Significant age-related declines were observed in the Composite Score (CS: rs = −0.532, *p* < 0.001), Visual Score (VIS: rs = −0.530, *p* < 0.001), and Vestibular Score (VEST: rs = −0.430, *p* = 0.002). ANOVA revealed significant inter-group differences in Equilibrium Scores (SOT2: *p* = 0.012; SOT3: *p* = 0.017; SOT4: *p* < 0.001; SOT5: *p* = 0.009; SOT6: *p* = 0.002) and CS (*p* = 0.001). Sensory scores showing parallel reductions (VIS: *p* < 0.001; VEST: *p* = 0.020; Preference, PREF: *p* = 0.018).RWS results demonstrated strong negative age-correlations for Directional Control (coronal: rs = −0.521, *p* < 0.001; sagittal: rs = −0.630, *p* < 0.001) and coronal Movement Velocity (rs = −0.398, *p* = 0.004), supported by significant inter-group differences in both planes (coronal: *p* = 0.002; sagittal: *p* < 0.001).

**Conclusion:**

Postural control demonstrated a significant age-related decline in this cohort. Notably, individuals over 45 years exhibited a diminished capacity to integrate visual and vestibular information for complex balance tasks and visually guided motor control. This study provides essential normative data for clinical assessment in the Chinese population and underscores the need for population-specific benchmarks.

## 1 Introduction

Postural control, a fundamental human motor function, comprise two key components: Postural orientation: the active regulation of trunk and head alignment relative to gravitational forces, support surfaces, visual environment, and internal reference frames; Postural stability: the maintenance of equilibrium through coordinated neuromuscular strategies in response to internal/external perturbations (1). Initial theories simplified it as a collection of balance reflexes driven by visual, vestibular, and proprioceptive inputs. However, subsequent studies revealed that postural control is a complex motor control processing, including sensory input, sensory integration and motor output (2). Postural control relies on the interaction of multiple subsystems, with six key factors crucial. (1) Biomechanical factors (3); (2) Movement Strategy (4); (3) Sensory strategy (5); (4) Spatial orientation (6); (5) Dynamic control; (6) Cognitive processing. Dysfunction in any subsystem may compromise postural stability. For instance, biomechanical factors (such as restricted ankle dorsiflexion ROM) will influence the posture control.

As a higher-order adaptive mechanism, sensory strategy comprises: Sensory integration—the CNS-mediated synthesis of afferent signals from somatosensory, visual, and vestibular modalities into a coherent spatial representation; Sensory reweighting—the CNS-mediated modulation of sensory weighting coefficients across visual, vestibular, and proprioceptive channels in response to contextual demands (7). This ability to re-weight sensory information allows for an increase or decrease in the influence of particular sensory inputs, depending on the availability or presence of other sensory sources (5). Generally, in healthy adults, postural stability during quiet stance predominantly relies on somatosensory inputs (≈70%), supplemented by vestibular (≈20%) and visual contributions (≈10%)—a weighting profile that is dynamically recalibrated under perturbed conditions (8).

Beyond environmental factors, sensory reweighting is significantly modulated by age-related neurodegenerative processes, characterized by reduced speed/accuracy in resolving sensory conflicts (e.g., visual-vestibular mismatch); impaired filtering of irrelevant sensory input; delayed adaptive recalibration to novel environments. Those will affect their perception of both their body and the surrounding environment (9, 10), which can lead to a deterioration in postural control and stability. Specifically, the weakening of sensory inputs—such as vision, somatosensory, and the vestibular system—compromises the body's ability to maintain balance.

The age-related decline in sensorimotor integration results from a hierarchical cascade: degenerative changes in peripheral proprioceptors and spinal interneurons degrade the fidelity and timing of sensory signals, which in turn triggers maladaptive cortical compensation (e.g., broader recruitment of motor areas). This multilevel degradation, from impaired signal acquisition to inefficient central processing, not only underlies poor sensory conflict resolution but also directly compromises motor output. This is behaviorally manifested as delayed compensatory stepping, reduced stability margins, and elevated sway entropy (11–15). Consequently, these functional declines significantly heighten the risk of falls—a leading cause of accidents among older adults, with approximately one-third of this population at increased risk (12, 16, 17). Therefore, precisely measuring the age-related decline in sensory reweighting capacity—a core postural control mechanism—is essential for developing targeted fall prevention strategies.

Conventional clinical tools (e.g., Berg Balance Scale, Y-Balance Test), while valuable for gross functional screening, lack the granularity to quantify the underlying sensory integration mechanisms. Computerized dynamic posturography (CDP), particularly the Sensory Organization Test (SOT), addresses this gap by directly probing multisensory integration capacity through calibrated environmental perturbations (15, 18). Unlike force-plate-based assessments focused solely on center-of-pressure (CoP) sway metrics, SOT employs precisely calibrated environmental perturbations (support surface translation, visual surround tilt) to isolate and quantify the relative contributions of somatosensory, vestibular, and visual inputs to posture control (19). Through calculating equilibrium scores and sensory ratios, SOT objectively characterizes sensory reweighting efficacy—a neural process inaccessible to traditional biomechanical tools. With advancements in virtual reality (VR) technology, the CDP has undergone iterative enhancements, leading to the development of the next-generation Bertec^®^ Balance Advantage (Bertec Incorporated, Columbus, OH, USA) CDPIVR device, which features an advanced dome design capable of encompassing the full range of vision. This evolution in technology not only improves the accuracy of balance assessments but also enhances the overall testing experience (20).

However, the interpretation of CDP/SOT results relies on population-specific normative data. Previous studies have primarily established such norms for American and European cohorts (19, 20). The generalizability of these Western norms to Chinese adults remains unverified—a critical gap given potential ethnocultural differences in lifestyle, physical activity patterns, and sensory reweighting strategies that may influence postural control. Disparities in postural control and fall risk are likely present among different racial and ethnic populations (16). Such differences may be attributed not only to geographic factors but also to culturally influenced underlying mechanisms. For instance, studies have shown that the fall rate among Chinese Australians is higher than that among Chinese individuals living in Asia. Interestingly, this suggests that protective behaviors against fall risk might be diminished after migration (21).

Therefore, establishing a normative database for healthy Chinese adults is not only necessary for accurate clinical assessment in this population but also presents a novel opportunity to investigate the potential influence of cultural and environmental factors on postural control. Several researchers have investigated normative data for local populations across different age groups and genders (19, 20). Previous studies have focused on American and European populations, while normative data exist for Western populations. Therefore, this study aims to: (1) Establish the normative SOT database for healthy Chinese adults across the lifespan (20–80 years); (2) Quantify cross-regional disparities by comparing our cohort with published Western data, thereby providing ethnicity-specific benchmarks for clinical assessment and fall risk.

## 2 Methods

This study is a cross-sectional study, which has been approved by the Ethics Committee of Beijing Sport University (2023242H) and has been registered with the Chinese Clinical Trial Registry under the registration number ChiCTR2300076578. All participants provided written informed consent in accordance with the Declaration of Helsinki.

### 2.1 Participants

This study was conducted at the Sports Rehabilitation Medicine Center of Beijing Sport University, where 60 Chinese nationals were recruited through various methods, including poster and online recruitment. Ultimately, 48 participants completed the entire testing process. Age groups were divided at 15-year intervals (group A: 18–30 years old, group B: 31–45 years old, group C: 46–60 years old, and group D: 61–75 years old) based on the critical postural control decline threshold at age 45 and the WHO-defined elderly boundary at age 60 (22, 23).

#### 2.1.1 Inclusion criteria

1) Eligible participants are Chinese adults aged 18–75 years.
2) Able to stand ≥30 s without upper limb support and walk ≥20 min unaided.
3) Berg Balance Scale score ≥41.
4) Corrected visual acuity ≥1.0 with intact visual fields (Logarithmic visual acuity chart, GB11533-−1989).
5) Participants must provide informed consent in writing and demonstrate the ability to complete all study assessments.

#### 2.1.2 Exclusion criteria

1) Neurological disorders: stroke, Parkinson's, Alzheimer's, epilepsy, multiple sclerosis, spinal cord injury.
2) Neurological and structural abnormalities: history of brain surgery, presence of brain tumors, or peripheral neuropathy.
3) Sensory or vestibular impairments: presence of visual or auditory impairments, vertigo, vestibular disorders, or severe postural control deficits.
4) Medications: psychoactive drugs, vestibular suppressants, or benzodiazepines within 3 months.
5) Musculoskeletal conditions: any musculoskeletal or rheumatoid disorders that impair the range of motion in the lower extremities.
6) Systemic diseases: uncontrolled hypertension (>150/100 mmHg), Uncontrolled diabetes (HbA1c > 6.5%), Cardiac dysfunction (NYHA ≥ III) or severe angina (CCS ≥ III) and any serious complications.

### 2.2 Procedure

**Figure 1:**
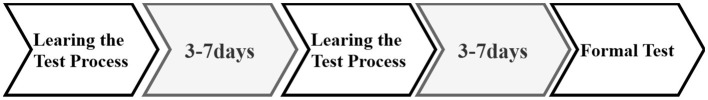
Flow chart.

To mitigate practice effects documented in CDP protocols (24), all participants completed two familiarization trials on the Bertec^®^ system prior to data collection. Formal test data were exclusively obtained during the third session. At the initial visit, after detailed explanation of experimental procedures and risks, written informed consent was obtained. Subsequently, the first familiarization trial commenced, the process including SOT and Rhythmic Weight Shift Test (RWS) (Figure 1).

**Figure 2:**
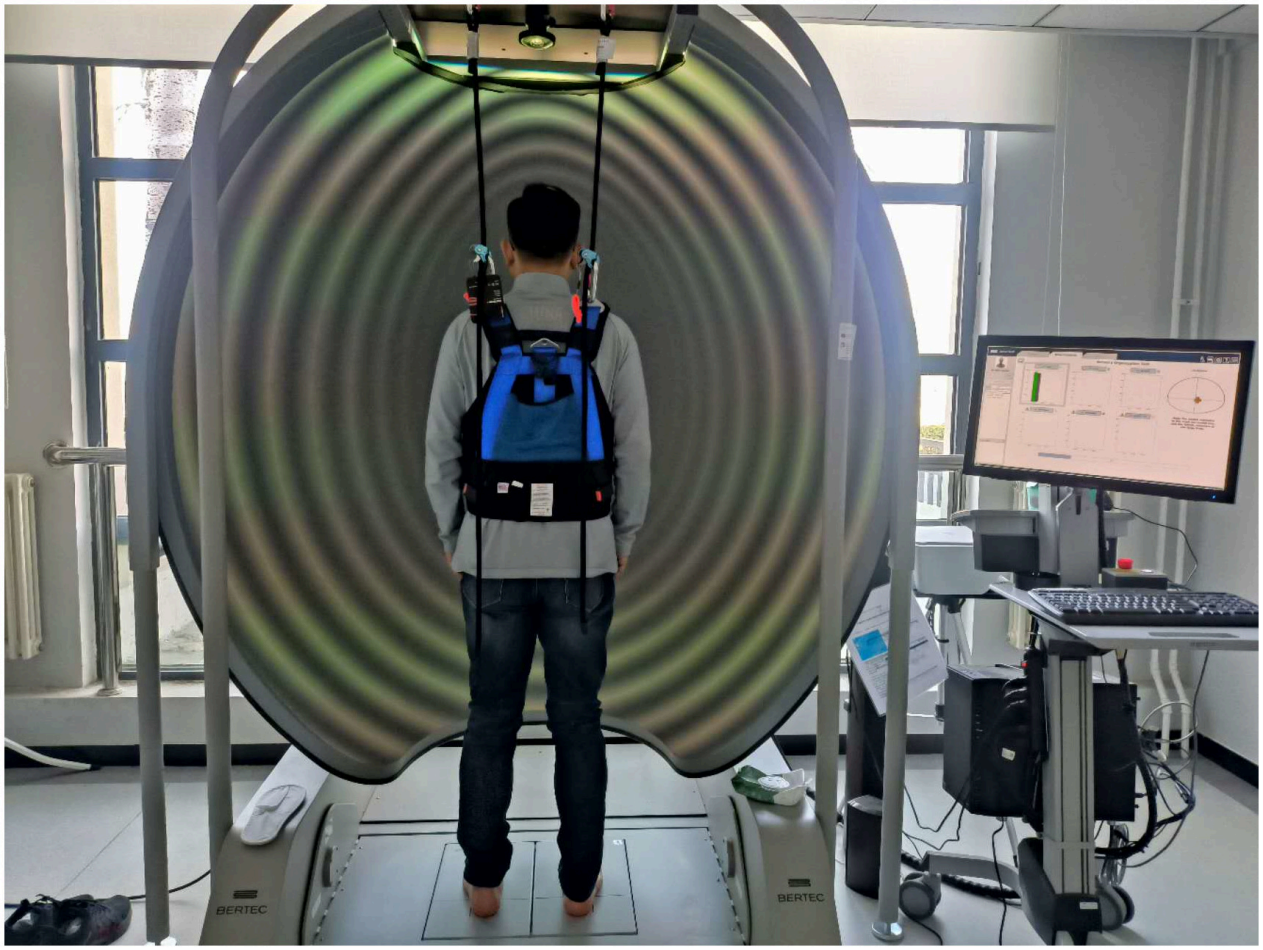
Laboratory layout and infrastructure.

Participants stood barefoot on the force platform in a standardized position. A safety harness—suspended from the dome mount—was secured at the waist to prevent falls without restricting movement (Figure 2). An experimenter stood behind the participant to intervene if necessary. In actual testing, ambient light in the lab is controlled to ≤ 5 lux using blackout curtains and switched ceiling lights to minimize visual distractions. All instructions were delivered in standardized Mandarin by the same experimenter:

- *SOT*: maintain balance while fixating straight ahead.
- *RWS:* rhythmically shift your center of gravity laterally to track the green ball's trajectory.

### 2.3 Outcome measures

There were two postural control tests involved in this study, Sensory Organization Test (SOT) and Rhythmic Weight Shift (RWS). The SOT composite score (CS) was selected as the primary outcome given its recognized validity in tests of postural control. The remaining indicators serve as secondary outcomes.

#### 2.3.1 Sensory organization test (SOT)

Participants were required to complete 18 trials, three trials of six conditions (Table 1). After completing the SOT, several scores were generated: the Equilibrium Score (ES) represents the difference between the participant's anterior and posterior center-of-pressure sway and the theoretical stability limit, defined as a maximum peak-to-peak sway of 12.5° [8°forward and 4.5° backward]. It is expressed as a percentage, ranging from 0% to 100%, with 100% indicating full stability (no sway) and 0% indicating fall. The Composite Score (CS) is a weighted average of the equilibrium score of all six conditions. Sensory Ratio Score (SS): is a set of systematic average scores help identify specific impairments in the sensory system. It includes separate scores for visual (VIS), somatosensory (SOM), and vestibular (VEST) inputs, along with the preference (PREF)ratio score. The SOM (SOT 2/SOT 1), VIS (SOT 4/SOT 1), and VEST ratio (SOT 5/SOT 1) scores were used to assess the ability to maintain postural control using each sensory system. The PREF (SOT 3 + 6/SOT 2 + 5) score evaluates an individual's ability to resist and counteract misleading visual information while maintaining postural control (25).

**Table 1:** Sensory organization test (SOT) conditions.

**Condition**	**Platform**	**Visual**
SOT 1	Platform fixed	Eyes open, background fixed
SOT 2	Platform fixed	Eyes closed, background fixed,
SOT 3	Platform fixed	Eyes open, background sway referenced
SOT 4	Dynamic platform	Eyes open, background fixed
SOT 5	Dynamic platform	Eyes closed, background fixed
SOT 6	Dynamic platform	Eyes open, background sway referenced

#### 2.3.2 Rhythmic weight shift (RWS)

During the RWS assessment, participants are instructed to voluntarily shift their weight at specified paces (1, 2, and 3 s) in either the anterior-posterior or lateral directions. The center of gravity sway velocity is measured in degrees per second and compared to normative scores from healthy individuals, based on the selected pace and direction. The primary objective is to evaluate the patient's ability to rhythmically control their center of pressure across different paces and directional shifts. Directional Control (DCL) is a measure of how straight the movement is along the targeted axis (Left/Right or Front/Back). The slow, medium and fast oscillations and the combined control scores are denoted as DCLS, DCLM, DCLF and DCLC. Movement Velocity (MVL) indicates how fast the patient moved between the two targets. Oscillating velocities are expressed as MVLS, MVLM, MVF and MVLC.

### 2.4 Statistics analysis

Statistical analyses were performed using jamovi (version 2.3.28). The normality of continuous demographic variables (age, height, weight, BMI) was assessed with the Shapiro-Wilk test, and homogeneity of variances was evaluated using Levene's test. The association between age and postural control parameters was first analyzed with Spearman's rank correlation. For group comparisons, either standard one-way ANOVA (Fisher's ANOVA) followed by Tukey's HSD *post hoc* test, or Welch's ANOVA followed by the Games-Howell *post hoc* test, was employed based on whether the assumptions of normality and homogeneity of variances were met. Categorical variables (e.g., sex) were analyzed with Pearson's chi-square test. Data from the SOT and RWS are presented as mean ± standard deviation. Additionally, one-sample *t*-tests (for normally distributed data) or Wilcoxon signed-rank tests (for non-normal data) were used to compare our SOT composite scores against existing Danish normative values (20).

## 3 Results

### 3.1 Subjects

Sixty participants were initially enrolled through community posters and offline recruitment channels. After screening, 48 completed all study protocols. Exclusions comprised:

- Five individuals with prior surgical history (orthopedic/neurological interventions).
- One older adult failing to complete testing due to fatigue.
- Six voluntary withdrawals (personal reasons).

Among the final cohort (*n* = 48), eight participants had corrected myopia (Corrected visual acuity ≥ 1.0 LogMAR with glasses), and eight reported stable chronic conditions:

- Controlled hypertension (*n* = 5, BP < 140/90 mmHg on medication);
- Type 2 diabetes (*n* = 2, HbA1c < 6.5%);
- Bronchial asthma (*n* = 1, FEV1 > 80% predicted).

No medication-related adverse effects (e.g., dizziness or hypotension) were observed during testing, suggesting negligible pharmacological interference with postural control measures.

### 3.2 Demographics

The demographic information is summarized in Table 2. In addition to age (*p* < 0.01), these four groups also differed significantly in terms of height (*p* = 0.009), and a *post hoc* test revealed that subjects in Group A were significantly taller than those in Groups C and D. Postural control data in this study have been standardized according to height, so comparisons between groups are valid and meaningful.

**Table 2:** Demographic characteristics by age group.

**Parameter**	**Group A (*n* = 12)**	**Group B (*n* = 12)**	**Group C (*n* = 12)**	**Group D (*n* = 12)**	***p*-value**
Sex (M/F)	6/6	7/5	5/7	6/6	0.766
Age (y)^*^	23.31 ± 2.18	36.89 ± 4.48	55.46 ± 2.4	63.55 ± 3.91	< 0.001^***^
Height (m)^*^	1.74 ± 0.09	1.68 ± 0.10	1.63 ± 0.10	1.62 ± 0.08	0.009^**^
Weight (kg)	69.34 ± 12.14	69.44 ± 13.82	62.38 ± 11.47	62.18 ± 10.04	0.265
BMI (kg/m^2^)	22.85 ± 2.47	24.30 ± 2.78	23.27 ± 2.53	23.83 ± 3.61	0.617
Exercise Habit(n)	7/12	4/12	4/12	5/12	0.122
Vision Correction(n)^*^	6/12	0	0	2/12	0.013^*^
Medical History(n)	0	1/12	3/12	4/12	0.215

^*^p<*0.05*, ^**^p<*0.01*, ^***^p<*0.001; BMI, body mass index*.

### 3.3 Aging-related postural control task sensory integration ability

This study identified significant age-related declines in postural control and sensory integration across age groups. Spearman's rank correlation analysis demonstrated strong negative associations between age and CS (*rs* = −0.532, *p* < 0.001), VIS, (*rs* = −0.530, *p* < 0.001)and VEST, (*rs* = −0.430, *p* = 0.002) (Table 3). Notably, more challenging SOT conditions (SOT4–6) exhibited steeper age-dependent deterioration (*rs* = −0.589 to −0.478, *p* < 0.001), whereas no significant age effects were observed for simple postural control tasks (SOT1–3) and SOM.

**Table 3:** Spearman rank correlation analysis between age and sensory integration test.

**Parameter**	**Spearman correlation coefficient (*rs*)**	**Degrees of freedom**	***p*-value**	**Number of cases**
SOT1	−0.155	46	0.279	48
SOT2	−0.189	46	0.185	48
SOT3	−0.173	46	0.224	48
SOT4^***^	−0.589	46	< 0.001	48
SOT5^***^	−0.468	46	< 0.001	48
SOT6^***^	−0.478	46	< 0.001	48
CS^***^	−0.532	46	< 0.001	48
SOM	−0.133	46	0.352	48
VIS^***^	−0.530	46	< 0.001	48
VEST^**^	−0.430	46	0.002	48
PREF	−0.142	46	0.319	48

^*^p<*0.05*, ^**^p<*0.01*, ^***^p<*0.001*.

SOT, sensory organization test; CS, composite score; SOM, somatosensory; VEST, vestibular; PREF, preference of visual; rs, Spearman's rank correlation coefficient. Vestibular; PREF, preference of visual; rs, Spearman's rank correlation coefficient.

**Figure 3:**
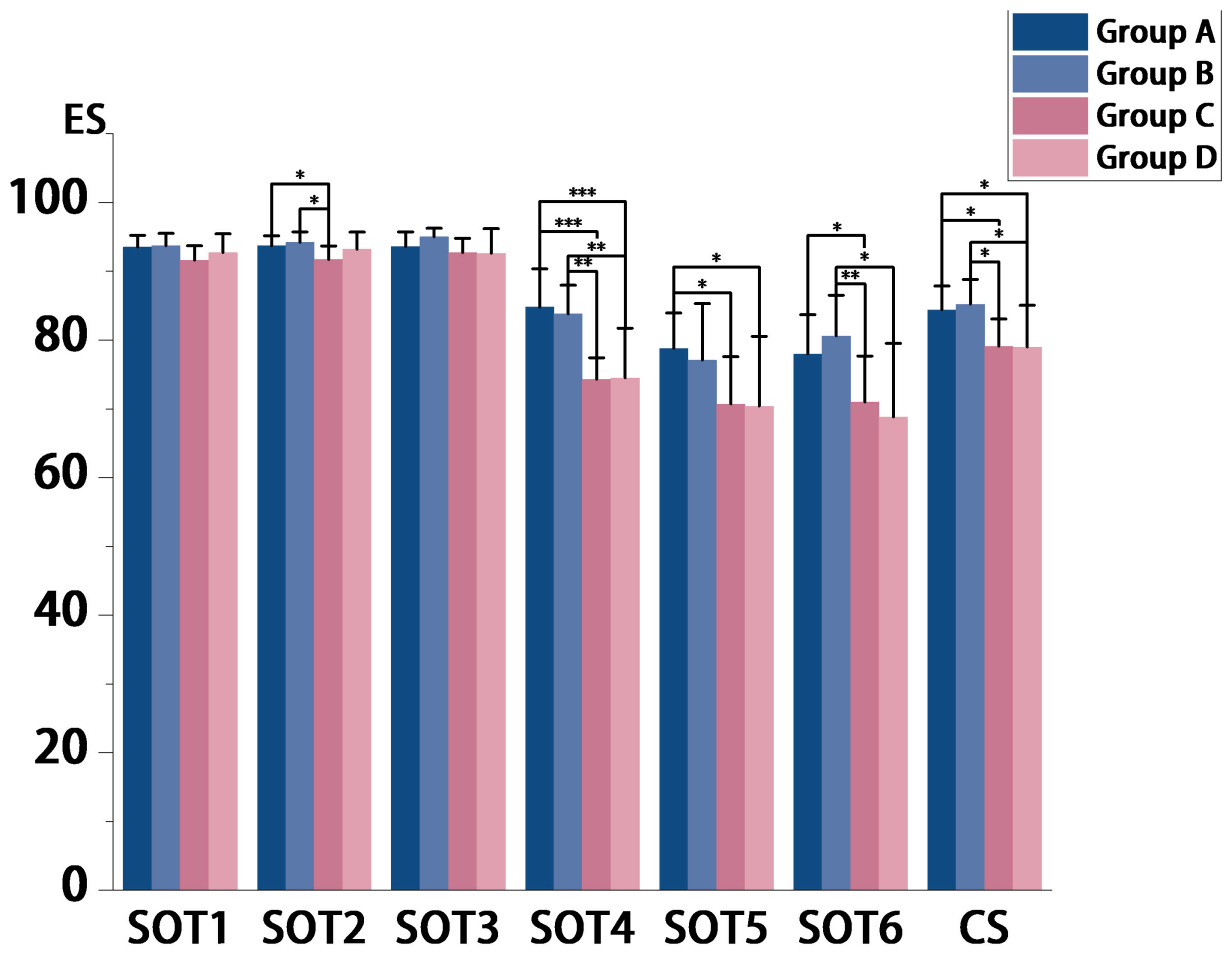
*p < 0.05,**P < 0.01,***p < 0.001; ES, Equilibrium score; SOT, Snesory organization test; CS, Composite score.

ANOVA revealed significant between-group differences in SOT (Table 4), particularly for dynamic balance tasks (SOT2-SOT6) and CS (SOT2: *p* = 0.012; SOT3: *p* = 0.017; SOT4: *p* < 0.001; SOT5: *p* = 0.009; SOT6: *p* = 0.002; CS: *p* = 0.001). As detailed in Figure 3, *post-hoc* analyses indicated that younger groups (A and B) consistently demonstrated superior postural stability compared to middle-aged and older groups (C and D). Specifically: In SOT2, Group C scored significantly lower than both Group A (*p* = 0.030) and Group B (*p* = 0.018). In SOT4, Groups C and D exhibited marked reductions relative to Groups A (both *p* < 0.001) and B (*p* = 0.005 and *p* = 0.008, respectively). In SOT5, Group A outperformed both Group C (*p* = 0.029) and Group D (*p* = 0.030). The Composite Score (CS) showed a similar pattern, with Group A significantly higher than both Group C (*p* = 0.010) and Group D (*p* = 0.012), and Group B outperforming Group C (*p* = 0.010) and Group D (*p* = 0.011). For SOT6, which was analyzed using the Games-Howell test due to heteroscedasticity, Groups A and B maintained a significant advantage over Groups C (*p* = 0.025 and *p* = 0.009) and D (*p* = 0.022).

**Table 4:** SOT and RWS score statistics for each group.

**SOT**	**Group A**		**Group B**		**Group C**		**Group D**		**Welch's**		**Fisher's**	
	**Mean** ±**SD**	**95% CI**	**Mean** ±**SD**	**95% CI**	**Mean** ±**SD**	**95% CI**	**Mean** ±**SD**	**95%CI**	***f*** **-value**	***p*** **-value**	***f*** **-value**	***p*** **-value**
C1	93.5 ± 1.71	92.6–94.5	93.7 ± 1.80	92.4–95.1	91.6 ± 2.11	90.4–92.8	92.7 ± 2.73	91.0–94.5	2.972	0.052		
C2^*^	93.7 ± 1.46	92.9–94.5	94.2 ± 1.53	93.0–95.4	91.7 ± 1.97	90.6–92.8	93.2 ± 2.50	91.7–94.8			4.077	0.012
C3^*^	93.6 ± 2.11	92.5–94.8	95.0 ± 1.24	94.0–95.9	92.7 ± 2.10	91.5–93.9	92.6 ± 3.59	90.3–94.9	4.071	0.017		
C4^***^	84.8 ± 5.54	81.8–87.7	83.8 ± 4.18	80.8–87.0	74.3 ± 3.12	70.2–78.4	74.5 ± 7.22	69.9–79.1			10.956	< 0.001
C5^**^	78.8 ± 5.12	76.1–81.0	77.1 ± 8.20	70.8–83.4	70.7 ± 6.89	66.7–74.7	70.4 ± 10.14	63.8–77.0			4.320	0.009
C6^**^	78.0 ± 5.67	74.9–81.0	80.6 ± 5.91	76.1–85.2	71.0 ± 6.69	67.1–74.8	68.8 ± 10.71	62.0–75.6	6.550	0.002		
Composite^**^	84.4 ± 3.46	82.5–86.2	85.2 ± 3.60	82.5–88.0	79.1 ± 3.96	76.9–81.4	79.0 ± 6.05	75.2–82.8	7.542	0.001		
SOM	100.4 ± 1.63	99.6–101	99.8 ± 2.59	97.8–102	100.0 ± 1.92	98.9–101	99.8 ± 4.20	97.1–102			0.195	0.899
VIS^***^	90.4 ± 5.83	87.3–93.5	88.8 ± 5.24	84.8–92.8	81.3 ± 7.27	77.1–85.5	80.5 ± 6.96	76.1–84.9			8.222	< 0.001
VEST^*^	84.1 ± 5.63	81.1–87.1	81.4 ± 8.66	74.8–88.1	76.8 ± 6.89	72.8–80.8	76.6 ± 8.51	71.2–82.0			3.618	0.020
PREF^*^	99.6 ± 3.74	97.6–102	103.2 ± 2.68	101–105	100.9 ± 4.58	98.3–104	98.3 ± 4.58	95.3–101	4.054	0.018		
**RWS_LR**												
DCLS^*^	83.2 ± 4.67	80.7–85.7	81.0 ± 7.62	75.1–86.9	76.5 ± 6.36	72,8–80.2	74.8 ± 11.1	67.3–82.2			3.611	0.020
DCLM	84.6 ± 6.60	81.1–88.1	87.6 ± 2.77	85.4–89.7	83.8 ± 4.68	81.1–86.5	83.3 ± 4.82	80.1–86.6	2.972	0.051		
DCLF^***^	92.8 ± 2.32	91.5–94.0	90.3 ± 2.85	88.1–92.5	87.5 ± 4.13	85.1–89.9	84.5 ± 5.83	80.6–88.4	10.523	< 0.001		
DCLC^**^	86.8 ± 3.69	84.9–88.8	86.3 ± 3.58	83.5–89.1	82.6 ± 4.27	80.2–85.1	80.9 ± 5.30	77.3–84.4			5.707	0.002
MVLS	3.41 ± 0.475	3.15–3.66	3.33 ± 0.442	2.99–3.67	3.51 ± 0.812	3.05–3.98	3.29 ± 0.516	2.95–3.64			0.333	0.801
MVLM	4.86 ± 0.684	4.50–5.23	5.01 ± 0.757	4.43–5.59	5.01 ± 0.934	4.47–5.55	4.77 ± 0.703	4.30–5.24	0.246	0.863		
MVLF^*^	9.10 ± 1.44	8.33–9.87	8.73 ± 2.44	6.86–10.6	7.91 ± 3.33	5.99–9.84	6.53 ± 2.31	4.97–8.08	3.538	0.032		
MVLC	5.78 ± 0.793	5.36–6.20	5.69 ± 0.914	4.99–6.39	5.46 ± 1.52	4.59–6.34	4.87 ± 0.930	4.25–5.50			1.649	0.191
**RWS_FB**												
DCLS	82.1 ± 6.98	78.4–85.8	73.4 ± 13.5	63.1–83.8	71.6 ± 14.1	63.5–79.8	72.7 ± 12.5	64.3–81.1	3.513	0.034		
DCLM^***^	84.5 ± 4.73	82.0–87.0	80.8 ± 7.55	75.0–86.6	73.8 ± 8.85	68.6–78.9	74.0 ± 9.71	67.5–80.6	7.433	0.001		
DCLF^***^	90.8 ± 2.80	89.3–92.3	85.8 ± 5.10	81.9–89.7	82.4 ± 6.27	78.8–86.0	82.2 ± 5.58	78.5–86.0	12.528	< 0.001		
DCLC^***^	85.8 ± 3.73	83.8–87.8	80.0 ± 7.21	74.5–85.6	75.9 ± 7.89	71.4–80.5	76.3 ± 6.40	72.0–80.6			9.306	< 0.001
MVLS	1.93 ± 0.529	1.64–2.21	2.09 ± 0.575	1.65–2.53	2.32 ± 0.668	1.94–2.71	2.09 ± 0.802	1.55–2.63	1.008	0.449		
MVLM	2.68 ± 0.543	2.39–2.97	3.08 ± 0.674	2.56–3.60	2.94 ± 0.631	2.58–3.31	2.91 ± 0,931	2.28–3.53	0.917	0.449		
MVLF	5.21 ± 0.734	4.82–5.60	5.38 ± 1.10	4.54–6.22	4.74 ± 1.54	3.85–5.63	4.33 ± 1.24	3.50–5.16	1.851	0.169		
MVLC	3.27 ± 0.474	3.02–3.53	3.51 ± 0.641	3.02–4.00	3.34 ± 0.802	2.87–3.80	3.10 ± 0.888	2.50–3.70	0.503	0.685		

^*^p < 0.05, ^**^p < 0.01, ^***^p < 0.001.

**Figure 4:**
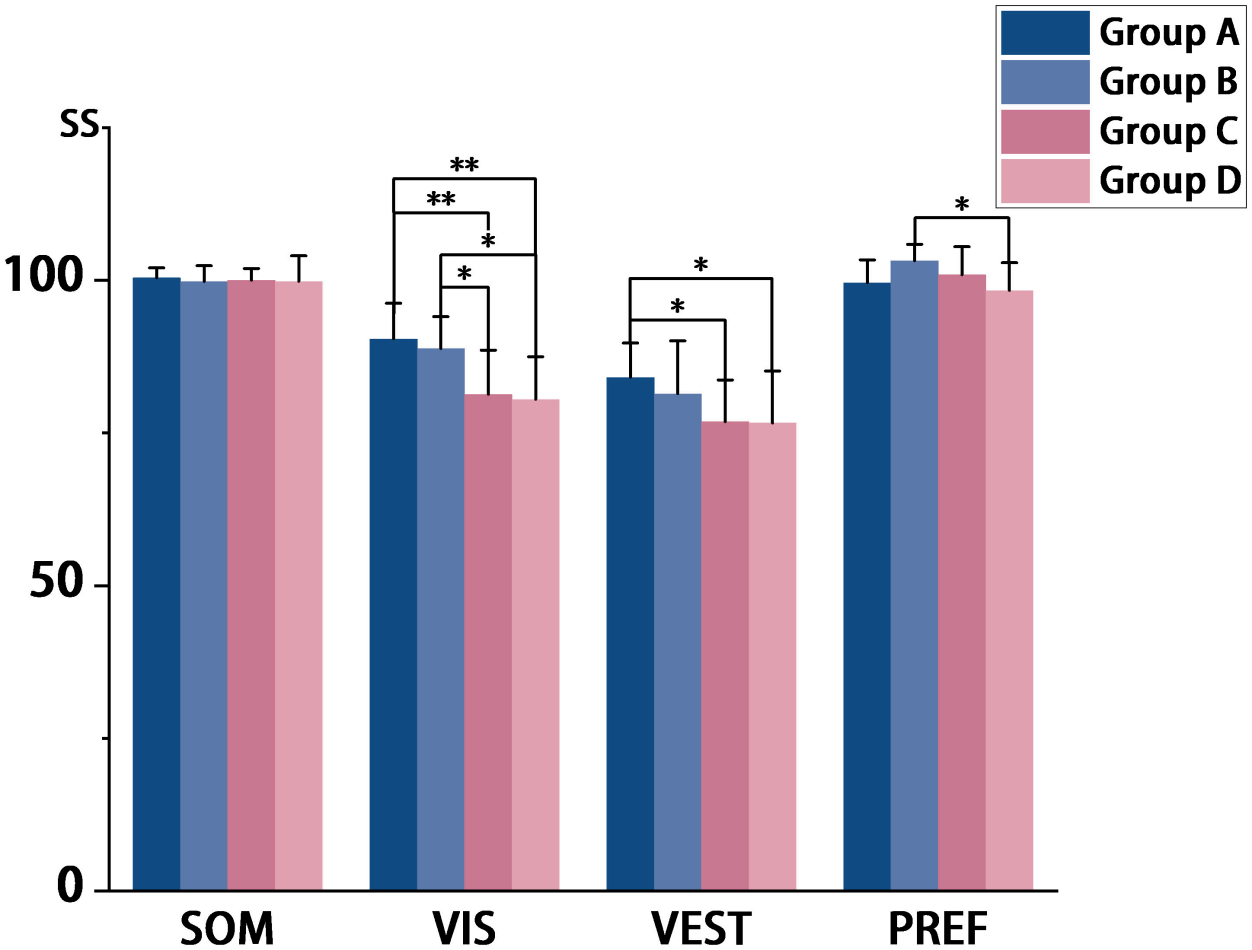
*p < 0.05,**P < 0.01,***p < 0.001; SS, Sensory Ratio score; SOM, Somatosensory; VIS, Vestibular; PREF, Preference of Visual.

Significant differences were also observed in sensory ratio scores, as summarized in Figure 4. The Visual Score (VIS) showed Group A scoring higher than Group D (*p* = 0.001), and Group B outperforming both Group C (*p* = 0.043) and Group D (*p* = 0.027). The Vestibular Score (VEST) was higher in Group A than in Group D (*p* = 0.044), and the Preference Score (PREF) was elevated in Group B compared to Group D (*p* = 0.037).

### 3.4 Aging-related visually guided postural control abilities

**Figure 5:**
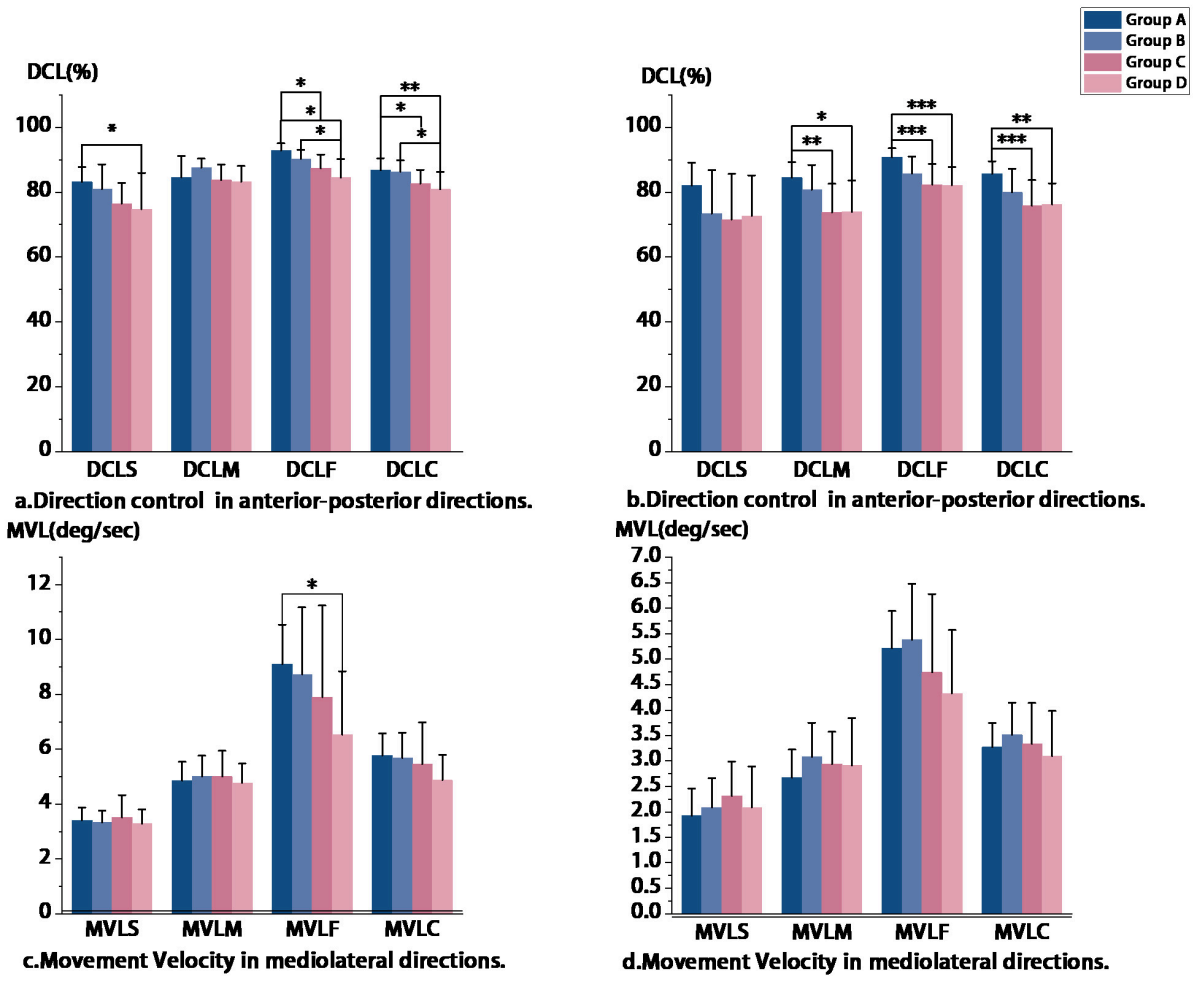
*p < 0.05,**P < 0.01,***p < 0.001; RWS-LR, Rhythmic Weight Shift in the left-right direction; RWS-FB, Rhythmic Weight Shift in the forward-back direction; MVLS, Slow Movement Velocity; MVLM, Medium Movement Velocity; MVLF, Fast Movement Velocity; MVLC, Composite Movement Velocity; DCLS, Slow Direction Control; DCLM, Meduim Direction Control; DCLF, Fast Direction Control; DCLC, Composite Direction Control.

RWS results indicated poorer directional control in both the mediolateral (*p* = 0.002) and anteroposterior (*p* < 0.001) directions in Groups C and D compared to the younger groups. The variance is primarily observed in the DCL (Figure 5), both in the mediolateral and anterior-posterior directions. In the mediolateral test, significant Intergroup differences were observed in DCLS (*P* = 0.020), DCLF (*P* < 0.001), DCLC (*P* = 0.002), and MVLF (*P* = 0.032). Tukey's HSD *post hoc* test revealed significant differences in DCLC between Group A and Group C (*p* = 0.044), Group A and Group D (*p* = 0.004), Group B and Group D (*p* = 0.032). In the anteroposterior test, significant intergroup differences were identified in DCLS (*P* = 0.034), DCLM (*P* = 0.001), DCLF (*P* < 0.001), and DCLC (*P* < 0.001). However, no intergroup differences in MVL were observed. Following this, Tukey's HSD *post hoc* test was conducted. For DCLC, significant intergroup differences were noted between Group A and Group C (*P* < 0.001), Group A and Group D (*P* = 0.002).

### 3.5 Comparison with Danish normative data

To explore potential cross-cultural variations, an exploratory comparative analysis was performed between the normative data from our Chinese cohort and existing data from a healthy Danish population (20). When comparing broadly age-matched groups (e.g., our Group A: 23.31 ± 2.18 years vs. the 20-29-year-old Danish cohort), notable variations were observed in several SOT parameters (Table 5). The most pronounced variations were identified among individuals in their 20s and 30s. These preliminary findings suggest that postural control characteristics may differ between these populations, highlighting the need for further investigation.

**Table 5:** Sensory organization test (SOT) of Chinese and Danish peers.

**Parameter**	**Group A vs 20-29**		**Group B vs 30-39**		**Group C vs 50-59**		**Group D vs 60-69**	
	**ES/SS**	***p*** **-value**	**ES/SS**	***p*** **-value**	**ES/SS**	***p*** **-value**	**ES/SS**	***p*** **-value**
SOT 1	94.1 ± 2	0.211	93.5 ± 2.3	0.189	93.1 ± 1.5	0.019^*^	92.8 ± 2	0.923
SOT 2	93 ± 1.8	0.079	93 ± 2.1	0.048^*^	91.5 ± 1.9	0.724	91.7 ± 2.3	0.055
SOT 3	92.2 ± 2.3	0.016^*^	92.7 ± 3.1	< 0.001^***^	90.6 ± 3	0.007^**^	90.3 ± 3.3	0.037^*^
SOT 4	77.6 ± 5.5	< 0.001^***^	79.2 ± 6.3	0.011^*^	75.4 ± 6.3	0.576	74 ± 6.6	0.085
SOT 5	68.6 ± 10	< 0.001^***^	69.5 ± 15.8	0.024^*^	68.5 ± 9	0.251	69 ± 8.6	0.654
SOT 6	69.7 ± 8	< 0.001^***^	68.6 ± 19.3	0.008^**^	67.3 ± 13.3	0.090	59 ± 13.9	0.009^**^
CS	79.3 ± 4.2	< 0.001^***^	79.8 ± 8.6	0.002^**^	78.1 ± 5.5	0.489	75.8 ± 5.1	0.094
SOM	98.8 ± 1.9	0.001^**^	99.5 ± 2.1	0.756	97.8 ± 2.1	< 0.001^***^	98.8 ± 2.6	0.450
VIS	82.7 ± 5.7	< 0.001^***^	84.8 ± 5.6	0.052	80.8 ± 6.8	0.806	79.7 ± 7.1	0.698
VEST	73.2 ± 10.1	< 0.001^***^	73.7 ± 16.2	0.028^*^	73.5 ± 9.1	0.098	74.2 ± 9.4	0.353
PREF	100.1 ± 6.3	0.574	99.2 ± 6	0.002^**^	98.8 ± 8.7	0.106	93 ± 7.5	0.005^**^

^*^p < 0.05, ^**^p < 0.01, ^***^p < 0.001.

ES, equilibrium score; SS, sensory ratio score; CS, composite score; SOM, somatosensory; VEST, vestibular; PREF, preference of visual

## 4 Discussion

This study may represent the third investigation into normative data for Bertec equipment. Building upon previous research, we optimized the experimental design by ensuring that subjects underwent the learning process more than twice, thereby evaluating both the SOT and the RWS in their entirety. This approach minimizes the learning effect and enables the tests to accurately reflect the participants' optimal performance.

### 4.1 SOT Equilibrium scores for different age groups

A significant negative correlation was observed between advancing age and the composite score (CS) (rs = −0.532). ANOVA results revealed significant differences among age groups for SOT condition 2, 4, 5, and 6, as well as for CS. *Post hoc* analyses indicated that ES were significantly higher in Groups A and B compared to Groups C and D, particularly in SOT condition 4, 5, and 6. Significant differences were observed between younger adults (Groups A and B) and middle-aged/older adults (Groups C and D). For instance, CS decreased from 85.2 in Group B to 79.1 in Group C (*p* = 0.01), suggesting a potential acceleration in postural control decline during the transition to middle age.

These findings are consistent with the recognized trajectory of accelerated postural control decline after age 45, driven by convergent degeneration in neuromuscular, articular, and sensory systems (26). Specifically, selective atrophy of fast-twitch muscle fibers—which deteriorate at ~3 × the rate of slow-twitch fibers after age 50—compromises rapid balance responses (22). In parallel, estrogen deficiency during menopause accelerates articular cartilage thinning and impairs joint proprioception (27). This decline represents a transition from adaptive compensation (where the CNS recruits ancillary neural resources before age 45) to progressive decompensation. It is important to note that our study was not powered to conduct sex-stratified analyses. However, the observed decline in middle age (Group C) warrants consideration of sex-specific factors in future research. For instance, in women, the menopausal transition involves endocrine alterations that can undermine musculoskeletal integrity (28, 29), potentially compounding age-related risks (30).

The most pronounced differences in ES emerged in SOT condition 4–6 compared to 1–3. SOT 4–6 employs a sway-referenced platform that disrupts proprioceptive input, thereby increasing reliance on visual and vestibular systems for stability maintenance. Consequently, older adults exhibit marked postural control deficits under proprioceptive perturbation—a critical sensory modality for detecting joint/limb kinematics and body orientation in space (31, 32). The impact of aging on proprioception has become a focal point in developmental and geriatric research (33). As established by Yang et al., proprioception serving as the foundation for motor control, demonstrates joint-specific developmental trajectories rather than generalized maturation (34). Proprioceptive acuity essential for postural control, particularly at the ankle, reaches functional maturity by age 3–4 years (35), and peaks in adulthood. As the primary weight-bearing interface during stance, the ankle complex provides the final neuromuscular adjustment for upright stability, rendering ankle proprioception clinically pivotal. Critically, evidence indicates progressive decline in ankle proprioceptive sensitivity after age 50 (34). Thus, age-related deterioration of proprioceptive function, especially at the ankle, is proposed as a key contributor to the reduced balance performance and composite scores observed in SOT conditions 4–6.

### 4.2 SOT Sensory ratio scores (SS) for different age groups

Sensory Scores (SS) reflect the capacity to utilize individual sensory systems for postural stability. Significant negative associations were observed between advancing age and both VIS (rs = −0.530, p < 0.001) and VEST (rs = −0.430, *p* = 0.002). ANOVA results indicated significant between-group differences in all scores except the SOM.

Vision is fundamental to postural control. Stable visual input from fixed environments reduces postural sway, whereas dynamic visual scenes may induce instability. When proprioception is compromised—such as during sway-referenced platform conditions or pathological states—visual dependence intensifies. This effect is often amplified in older adults, who may exhibit greater susceptibility to visual perturbations than younger individuals (36, 37). Consistent with this, our data showed lower VIS scores in middle-aged and older participants, suggesting a decline in the efficacy of utilizing visual information for balance with age. Crucially, visual reliance should not be conflated with VIS. Multiple studies report a paradoxical age-related phenomenon: a diminished ability to use visual input efficiently for balance may coincide with an increased weighting of visual information in sensorimotor processing (38). Ana Faraldo-García et al. documented a U-shaped trajectory, in which the contribution of vision to postural control declines to its lowest point between ages 40–49 before gradually increasing thereafter (39). Collectively, these findings suggest that older adults may exhibit heightened dependence on visual cues, underscoring the critical—and potentially increasingly dominant—role of vision in aging postural control systems.

Vestibular function typically begins to decline during middle age, though this deterioration often remains subclinical due to robust neural compensation. Comprehensive assessment usually requires specialized protocols—such as rotational chair testing—that quantify vestibular-ocular reflex (VOR) integrity by measuring extraocular muscle responses (40). Our SOT data suggest a progressive attenuation of vestibular contributions to postural stability beginning in midlife. Compromised vestibular processing may manifest as delayed transition movements (e.g., increased sit-to-stand latency) (41), highlighting the clinical value of functional assessments like the Timed Up and Go (TUG) test. Critically, while vestibular pathology can cause vertigo, age-related postural control decline rarely stems exclusively from vestibular dysfunction (40). Instead, it likely involves a multisystem failure including degraded sensory integration, impaired central processing, and compromised motor execution. This multifactorial etiology may explain why isolated vestibular interventions often yield limited functional improvement in geriatric populations.

Visual Preference (PREF) scores are derived from the formula (Condition 3 + Condition 6)/(Condition 2 + Condition 5), which compares balance performance when visual input is altered vs. absent. This score reflects the ability to suppress misleading visual information. It is widely recognized that as individuals age, especially middle-aged and older adults, they tend to rely more heavily on visual input to maintain balance (36). This increased reliance can make them more susceptible to being misled by inaccurate visual information, thereby potentially increasing fall risk. Moreover, the ability to resolve such sensory conflict is closely linked to cognitive function. Age-related neurological changes, such as brain atrophy and white matter lesions, can impair cognitive abilities, particularly those associated with the prefrontal lobes (42). Given that cognitive performance typically peaks around age 35 (43), the observed differences in PREF scores between Groups B and D in our cohort may be related to these age-related cognitive changes.

### 4.3 Age-related differences in visually guided motor control gains

The RWS task represents a simple fixed-speed visual tracking paradigm. Significant between-group differences were observed in DCL, particularly during fast-tracking conditions. For instance, in left-right fast oscillation (DCLF), Group A demonstrated better performance than Group D (92.8 ± 2.32 vs. 84.5 ± 5.83, *p* < 0.001), which is consistent with the expected superior motor control in younger adults.

The observed age-related decline in DCL, which reflects motor control precision, may be attributed to several interconnected mechanisms reported in the literature: (1) Peripheral degradation, including altered nerve conduction velocity and proprioceptive shifts; (2) Central atrophy, involving gray and white matter loss in motor cortices and associated network reorganization (44); (3) Neurochemical decline, such as dopaminergic depletion that can impair motor skills; (4) Reduced neural compensation efficiency, where over-recruitment of prefrontal-basal ganglia circuits may create a supply-demand imbalance vulnerable to age-related decline (45). These mechanisms collectively could help explain the reduced DCL performance in middle-aged and older adults during rapid oscillations, where precise control is paramount.

While DCL showed consistent age-related declines in both coronal and sagittal planes, the magnitude of decline differed biomechanically. Coronal plane control primarily relies on hip abductor/adductor synergy, whereas sagittal control is dominated by ankle strategies. The preferential atrophy of fast-twitch fibers in proximal musculature may accelerate coronal plane dysfunction (46). This is compounded by evidence suggesting ~30% greater hip joint shear forces during coronal vs. sagittal motion (47), which could accelerate articular cartilage wear and attenuate proprioceptive feedback. This biomechanical context aligns with the exclusive negative correlation between aging and coronal plane velocity found in our analyses and may underlie the stronger age-correlated decline in MVL observed in the coronal plane.

### 4.4 Comparison of China's and Denmark's SOT normative data

Compared to published Danish normative data (20), Groups A and B in our Chinese cohort demonstrated significantly higher SOT scores. This observed difference may be attributed to several factors. Methodologically, our protocol included ≥2 full practice trials prior to formal testing, which may have enhanced task familiarity and introduced procedural learning effects. Furthermore, a substantial proportion of our younger participants (36% in Group A, 29% in Group B) reported engaging in structured exercise regimens (>3 sessions/week), suggesting generally higher physical activity levels that could contribute to superior performance. It is also plausible that culturally influenced lifestyle factors play a role. For instance, whereas cycling culture in Denmark may promote sagittal-plane stability (48), the prevalent use of public transportation (e.g., buses and subways) in China could provide frequent, incidental exposure to multi-directional perturbations, potentially serving as a form of daily balance training in dynamic environments. In summary, the higher scores observed in our cohort are likely driven significantly by protocol-specific training effects and potentially higher baseline physical activity, rather than representing an inherent physiological advantage. These findings underscore the importance of standardizing acclimatization procedures and accounting for lifestyle factors in cross-cultural posturography research.

### 4.5 Clinical implications and future directions

The clinical implications of our findings are two-fold. First, the established normative data provide a crucial reference for clinicians to objectively assess sensory integration function in Chinese adults across different ages. By comparing a patient's performance against these benchmarks, particularly the sensory ratios that pinpoint reliance on visual, vestibular, or somatosensory inputs, practitioners can identify specific deficits beyond what traditional balance tests reveal. Second, the observed differences between our study and Western data underscore the necessity of using population-specific norms for accurate risk assessment. Employing Western criteria could potentially lead to either over- or under-estimation of fall risk in Chinese individuals. Therefore, this work provides the foundational evidence needed to develop targeted, evidence-based fall prevention strategies that are sensitive to the specific sensorimotor profile of the Chinese population.

### 4.6 Limitations

This study has several limitations. The modest sample size (*N* = 48) and the upper age limit of 72 years limit the generalizability of our findings, particularly regarding the oldest-old population. The non-random recruitment method may also introduce selection bias. Furthermore, detailed physical activity profiles and stricter health screening were not implemented; including these in future studies would help control for confounding variables. Finally, the acclimatization protocol (two practice trials) may have been insufficient for older adults, potentially attenuating the observed age-related differences. Therefore, our results should be interpreted as exploratory and might underestimate the true magnitude of postural control decline in the broader Chinese population.

## 5 Conclusion

In this sample, declining postural control was significantly associated with increasing age. Furthermore, individuals over 45 years of age in our cohort appeared to have a diminished capacity to utilize visual and vestibular information for maintaining stability during complex postural tasks and visually guided motor control.

## Data Availability

The raw data supporting the conclusions of this article will be made available by the authors, without undue reservation.
